# ‘Medusa head ataxia’: the expanding spectrum of Purkinje cell antibodies in autoimmune cerebellar ataxia. Part 2: Anti-PKC-gamma, anti-GluR-delta2, anti-Ca/ARHGAP26 and anti-VGCC

**DOI:** 10.1186/s12974-015-0357-x

**Published:** 2015-09-17

**Authors:** S. Jarius, B. Wildemann

**Affiliations:** Molecular Neuroimmunology Group, Department of Neurology, University of Heidelberg, Otto Meyerhof Center, Im Neuenheimer Feld 350, D-69120 Heidelberg, Germany

**Keywords:** Autoimmune cerebellar ataxia, Cerebellitis, Paraneoplastic cerebellar degeneration, Autoantibodies, Purkinje cells, Metabotropic glutamate receptor 1 (mGluR1) antibodies, Homer-3 antibodies, Anti-Sj, Inositol 1,4,5-trisphosphate receptor 1 (ITPR1, I3PR) antibodies, Carbonic anhydrase-related protein VIII (CARP VIII) antibodies, Protein kinase gamma (PKCγ) antibodies, Anti-Ca, Rho GTPase activating protein 26 (ARHGAP26, GRAF) antibodies, Glutamate receptor delta-2 (GluRδ2) antibodies, Anti-Yo, Cerebellar degeneration-related protein 2 (CDR2) antibodies, Cerebellar degeneration-related protein 2-like (CDR2L) antibodies, Purkinje cell antibody 2 (PCA-2), Anti-Tr, Delta notch-like epidermal growth factor-related receptor (DNER) antibodies, Anti-Nb, Anti-AP3B2, Neuronal adaptin-like protein (beta-NAP) antibodies, Voltage-gated calcium channel (VGCC) antibodies

## Abstract

Serological testing for anti-neural autoantibodies is important in patients presenting with idiopathic cerebellar ataxia, since these autoantibodies may indicate cancer, determine treatment and predict prognosis. While some of them target nuclear antigens present in all or most CNS neurons (e.g. anti-Hu, anti-Ri), others more specifically target antigens present in the cytoplasm or plasma membrane of Purkinje cells (PC). In this series of articles, we provide a detailed review of the clinical and paraclinical features, oncological, therapeutic and prognostic implications, pathogenetic relevance, and differential laboratory diagnosis of the 12 most common PC autoantibodies (often referred to as ‘Medusa head antibodies’ due their characteristic somatodendritic binding pattern when tested by immunohistochemistry). To assist immunologists and neurologists in diagnosing these disorders, typical high-resolution immunohistochemical images of all 12 reactivities are presented, diagnostic pitfalls discussed and all currently available assays reviewed. Of note, most of these antibodies target antigens involved in the mGluR1/calcium pathway essential for PC function and survival. Many of the antigens also play a role in spinocerebellar ataxia. Part 1 focuses on anti-metabotropic glutamate receptor 1-, anti-Homer protein homolog 3-, anti-Sj/inositol 1,4,5-trisphosphate receptor- and anti-carbonic anhydrase-related protein VIII-associated autoimmune cerebellar ataxia (ACA); part 2 covers anti-protein kinase C gamma-, anti-glutamate receptor delta-2-, anti-Ca/RhoGTPase-activating protein 26- and anti-voltage-gated calcium channel-associated ACA; and part 3 reviews the current knowledge on anti-Tr/delta notch-like epidermal growth factor-related receptor-, anti-Nb/AP3B2-, anti-Yo/cerebellar degeneration-related protein 2- and Purkinje cell antibody 2-associated ACA, discusses differential diagnostic aspects, and provides a summary and outlook.

## Introduction

Autoimmune cerebellar ataxia (ACA) is an important differential diagnosis in patients presenting with signs and symptoms of cerebellar disease. Alongside multiple sclerosis and acute disseminated encephalomyelitis, autoantibody-associated disorders of the central nervous system (CNS) are the most common cause of ACA. While ACA is a rare manifestation in some of those disorders, such as aquaporin-4 (AQP4) antibody-associated neuromyelitis optica (NMO), it is the most frequent or exclusive presentation in others. To date, around 30 different autoantibodies targeting brain antigens have been reported in patients with ACA, many of which are of paraneoplastic origin.

When tested by immunohistochemistry (IHC) using cerebellum tissue sections, some of these antibodies show a staining pattern resembling a Gorgon’s head, caused by binding of immunoglobulin G (IgG) to Purkinje cell (PC) somata and dendrites, and are therefore often referred to as ‘Medusa head’ antibodies. Most of these antibodies are involved in regulation of calcium homoeostasis in PCs.

In part 1 of this series of articles, we focused on anti-metabotropic glutamate receptor 1 (mGluR1)-, anti-Homer protein homolog 3 (Homer3)-, anti-Sj/inositol 1,4,5-trisphosphate receptor (ITPR1)- and anti-carbonic anhydrase-related protein VIII (CARP VIII)-associated ACA [1]. In the present, second part, we summarise the current knowledge on anti-protein kinase C gamma (PKCγ)-, anti-glutamate receptor delta-2 (GluRδ2)-, anti-Ca/RhoGTPase-activating protein 26 (ARHGAP26)- and anti-voltage-gated calcium channel (VGCC)-associated ACA.

## Anti-PKCγ

### Clinical, paraclinical and epidemiological features

Autoantibodies to PKCγ were first reported in 2006 in a 47-year-old male patient with paraneoplastic cerebellar degeneration (PCD), cerebrospinal fluid (CSF) pleocytosis and serum antibodies to PKCγ [2]. The patient’s exact clinico-neurological status and his brain MRI findings were not reported. A second patient presented with vertigo, vomiting, gait and limb ataxia, dysdiadochokinesia and dysarthria; CSF findings were normal; and MRI was not reported [3].

### Tumour association

Anti-PKCγ autoantibodies were discovered by screening a series of patients with PCD and non-small-cell lung cancer for unknown serum reactivities. While the non-small-cell lung cancer (NSCLC) of the index patient strongly expressed PKCγ and accordingly immunoreacted with the patient’s serum, NSCLC from patients without PCD did not express PKCγ at either RNA or protein level, strongly corroborating a paraneoplastic pathogenesis in this case [2, 4]. Neurological symptoms preceded tumour diagnosis by 2.5 months. In the second patient, tumour screening revealed a papillary adenocarcinoma of hepatobiliary origin infiltrating the liver.

### Outcome and prognosis

The index patient developed moderately severe disability. When last examined, he was unable to attend to his own bodily needs without assistance or to walk unassisted (Rankin score 4). He later died from cancer despite chemotherapy. The second patient was lost to follow-up after cancer therapy [3].

### Antigen

PKCγ is a brain neuron-specific member of the classical protein kinase C (PKC) family. Upon binding of cytosolic Ca^2+^ released by ITPR1 to its Ca^2+^-binding C2 domain, it is translocated to the plasma membrane, where it gets activated by diacylglycerol (DAG) binding to its C1 domain [5, 6]. PKC modulate the function of other proteins by phosphorylation of hydroxyl groups of threonine and serine amino acid residues and are involved in several signal transduction cascades.

PKCγ, which is expressed exclusively in neurons within the CNS, is thought to be involved in synaptic transmission, in particular in modulation of synaptic plasticity for long-term potentiation (LTP) and long-term depression (LTD) [5]. While PKC inhibitors block LTD in cultured rodent PCs, PKC activators mimic LTD [7, 8]. PKC have been implicated in desensitising mGluR1 by phosphorylation, which is thought to play a role in restitution of membrane potentials after action potential propagation, and activation of mGluR1 has been shown to generate oscillatory DAG, IP3 and calcium that respond to oscillations in the activation of PKC [9–12]. PKCγ was also reported to phosphorylate and block DAG-activated canonical transient receptor potential channels (TRPCs) [13, 14], resulting in reduced permeability for calcium ions [15]. PKCγ also modulate the function of GluR4-containing α-amino-3-hydroxy-5-methyl-4-isoxazolepropionic acid (AMPA) receptors by directly binding to GluR4 in the cerebellum; phosphorylation by PKC seems to target the receptor to the plasma membrane [16]. The activity of PKCγ, on the other hand, is indirectly regulated by DAGKη und DAGLα, which catalyses the hydrolysis of DAG [17]. Calcium and DAG binding recruits PKCγ to the plasma membrane, where it binds to membrane-linked receptors for activated C kinases (RACK) [18, 19]. Two isoforms of human PKCγ produced by alternative splicing are known.

### Immunohistochemistry

**Fig. 1:**
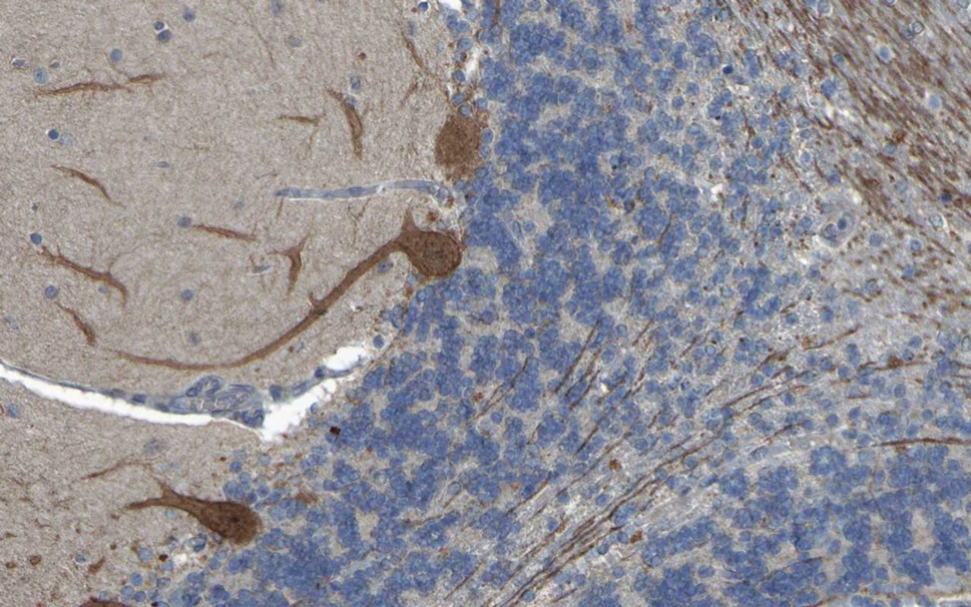
Expression of PKCγ in the human cerebellum as demonstrated by IHC (modified image from the *Human Protein Atlas* image database [20])

PKCγ expression is restricted to the CNS. Within the CNS, PKCγ is expressed at highest levels in the cerebellum (Fig. 1), followed by hippocampus and cerebral cortex [5, 20]. Using high-resolution immunogold cytochemistry of embedded PCs, staining of the plasma membrane of the dendrites, including the spines, with most intense staining in the PSD, cell body and, particularly, the initial segment of the PC axons, was observed [21]. Of note, two pools of PKCγ were distinguishable, a membrane-associated pool and a cytoplasmic pool located within 50 nm of the plasma membrane [21]. In addition, PKCγ was found to be enriched in the PC axon terminals in the dentate nuclei [21]. Organelle-related PKCγ was detected only on the *trans*-face of the Golgi apparatus.

**Fig. 2:**
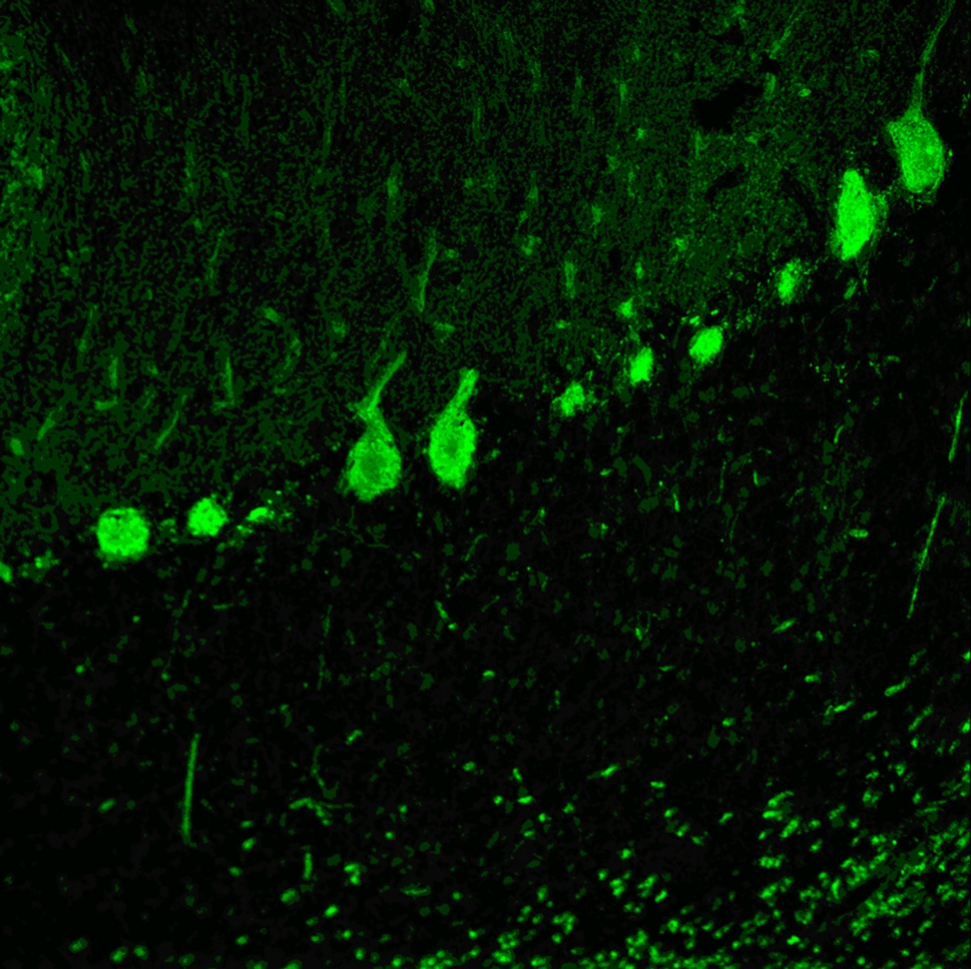
Binding of anti-PKCγ from a patient with ACA to a monkey cerebellum tissue section. The patient antibody was detected by use of a goat anti-human IgG secondary antibody labelled with Alexa Fluor® 488 (*green*)

In the two index patients, avidin–biotin immunoperoxidase IHC on frozen sections of paraformaldehyde-fixed rat cerebellum was used to detect anti-PKCγ [2, 3]; Sabater et al. used in addition human tissue [2]. The antibodies intensely labelled PC cytoplasm, dendrites and axons [2]. In addition, the plasma membrane of neurons in the deep cerebellar nuclei was outlined in a punctate manner corresponding to densely apposed synaptic terminals of Purkinje cell axons [2, 3]. By contrast, no reactivity was observed in non-CNS rat tissues [2]. Höftberger et al. reported binding of anti-PKCγ also to paraffin-embedded tissue [3]. Figure 2 shows staining of snap-frozen and formalin-fixed cerebellum tissue by anti-PKCγ as detected by indirect immunofluorescence.

### Antigen-specific assays

Anti-PKCγ has been shown to react with an 80-kilodalton (kDa) band in immunoblots of rat cerebellum [2, 3]. A commercial primate cerebellum blot was shown to contain PKCγ using a polyclonal antibody but has not been evaluated with patient serum [22]. So far, no PKCγ-specific cell-based assay (CBA) is available.

### CSF testing

No data on PKCγ levels in the CSF have been reported so far.

### Association with other autoantibodies

Sabater et al. did not find PKCγ immunoreactivity in the serum of 105 patients with anti-Hu (*n* = 50), anti-Yo (*n* = 30) or anti-CV2 (*n* = 25) antibodies [2].

### Pathogenetic relevance

The fact that PKCγ is an intracellular antigen located on the cytoplasmic side of the plasma membrane argues a priori against a role of antibody-dependent cell- or complement-mediated cytotoxicity. However, uptake of human IgG by PCs has been repeatedly described in independent studies [23–32]. Given the important role of PKCγ in regulating important signalling pathways, a functional impact of anti-PKCγ thus cannot be completely ruled out.

### Molecular genetics

Numerous mutations in the PKCγ gene have been found in patients with SCA14, which is characterised by slowly progressive, adult-onset cerebellar gait and limb ataxia, dysarthria and eye movement disturbances [33–42]. Some mutations were accompanied by moderate intellectual disability [42]. While mutations affecting the regulatory domain have been reported to be associated with a relatively pure form of SCA14 and onset in the third or fourth decade of life, mutations in the catalytic domain affect both children and adults and are associated with cognitive impairment in some cases [40, 43]. Selected PKCγ mutants show an increase instead of loss in kinase activity. This leads to aprataxin (APTX), a deoxyribonucleic acid (DNA) repair protein, being prevented from entering the nucleus due to phosphorylation and, in consequence, to increased oxidative stress-induced DNA damage and cell death [41]; mutations in the APTX gene underlie early-onset ataxia with oculomotor apraxia and hypoalbuminaemia [44]. Chen et al. found a reduction in immunoreactivity for ataxin-1 (ATXN1) in PC of patients with SCA14 due to PKCγ mutation [33]; on the other hand, loss of PKCγ may also contribute to PC pathology in SCA1, which is primarily caused by the expression of mutant ATXN1 [45]. PKCγ-deficient mice exhibit, alongside other symptoms, impaired motor coordination, probably caused by persistent multiple innervations of climbing fibres (CFs) on PC [5, 46]. Multiple innervations have also been observed in PKCγ mutant mice [47].

## Anti-GluRδ2

### Clinical, paraclinical and epidemiological features

Antibodies to GluRδ2 (immunoglobulin G [IgG] and M [IgM]) were first reported in 2004 in a 20-month-old child [48] and again in 2010 in two boys (18 months and 13 years of age, respectively) with cerebellar ataxia, one of whom was positive for GluRδ2-IgM instead of IgG [49, 50]. Clinical findings included dysmetria, dysdiadochokinesis, tremor, muscular hypotonia and gait ataxia.

Four additional patients, a 13-year-old girl with severe truncal ataxia [51] and three women aged 36, 45 and 59 years with ‘non-herpetic acute limbic encephalitis’ [52–55] were in addition positive for antibodies to the glutamate receptors epsilon2 (GluRε2), i.e. the *N*-methyl-d-aspartate receptor subtype 2B (NMDAR2B or NR2B), which have been described in patients with acute limbic encephalitis [52, 56–59]. In a young boy who had acute encephalitis with refractory, repetitive partial seizures, anti-GluRδ2 antibodies were reportedly present in addition to antibodies to GluRε2/NR2B and antibodies to the glutamate receptor zeta2 (GluRζ1) [60], i.e. the NMDA receptor subtype NMDAR1A or NR1A, the main target in anti-NMDAR receptor encephalitis [61].

MRI was normal in the index patient [48]. In the second patient, MRI revealed swelling (and later atrophy) of the left cerebellar hemisphere and obstructive hydrocephalus [49]. In the third patient, no cerebellar ataxia but meningeal enhancement was detected [50]; meningeal signs had been present also in patient 2. In a patient with anti-GluRδ2 and anti-GluRε2 antibodies, MRI findings normalised after plasma exchange (PEX) treatment [54].

In patient 2, lymphocytic CSF pleocytosis (404 cells/μl) was noted with negative oligoclonal bands (OCB). Lumbar puncture was normal in patient 3 at onset and demonstrated predominantly mononuclear pleocytosis but negative OCB later in the disease course. OCB were also lacking in the patient with anti-GluRδ2, anti-GluRε2 and anti-GluRζ1.

### Tumour association

No tumours have been reported so far in patients with anti-GluRδ2-associated cerebellar ataxia. Of note, however, all cases were preceded or accompanied by either infection or vaccination.

### Outcome and prognosis

In the index patient, intravenous immunoglobulins (IVIG) and intravenous methylprednisolone (IVMP) failed to ameliorate the symptoms; symptoms were still present 2 years later and were then accompanied by mental retardation [48]. The second patient recovered without any sequelae within 3 weeks following treatment with IVMP [49]. In the third patient, ataxia spontaneously and gradually improved, but worsened again following vaccination against mumps and varicella zoster. Interestingly, CSF neuron-specific enolase (NSE) levels correlated with the cerebellar symptoms in the index patient.

In those cases positive for anti-GluRδ2 and anti-GluRε2 and/or GluRζ1, cerebellar symptoms were persistent in the child (treatment status not reported) [51], while they completely disappeared in an adult patient after seven PEX treatments and around 2 months after onset [54]. In the remaining cases, limbic signs were predominant and either persisted [52] or were fatal within 2 weeks [53].

### Antigen

The protein termed glutamate receptor delta-2 (GluRδ2, also known as GluD2), encoded by the GRID2 gene, is nominally a member of the ionotropic glutamate receptor family of excitatory neurotransmitter receptors. However, as it is the case with GluRδ1, this classification is mainly based on sequence homology. It is still controversial whether GluRδ2 can in fact form functional ligand-gated ion channels in vivo [62]. While it does not recognise l-glutamate or glutamate analogues, the ligand-binding core of GluRδ2 binds neutral amino acids such as d-serine and glycine [63]. No functional incorporation of GluRδ2 into native ionotropic GluRs could be demonstrated [64], and obtaining ion channel function required ligand-binding domain transplantation from other glutamate receptors [65]. Thus, it has been proposed that the receptor might have other (e.g. modulatory) functions rather than serving as an ion channel [64, 66, 67]. Alternatively, GluRδ2 may be a ‘switchable’ receptor, the ionotropic functions of which may be controlled by yet uncharacterised proteins [65].

GluRδ2 is selectively expressed in PCs [68–70] and has been demonstrated to have an important role in long-term synaptic plasticity in the cerebellum and thus in motor learning [66, 67, 71, 72]. Recently, it was shown that GluRδ2 associates in PCs with mGluR1 and PKCγ, both of which have also been identified as target antigens in ACA (see above), as well as with the mGluR1/DAG- and PKCγ-regulated TRPC3, which is needed for mGluR1-dependent slow EPSCs [73]. Accordingly, mutations in GluRδ2 were associated with abnormal mGluR1-dependent synaptic transmission at parallel fibre (PF)/PC synapses, partly by increased surface expression of mGluR1 and TRPC3 and mGluR1-evoked inward currents [73]. It has been suggested that GluRδ2 interacts with mGluR1alpha in the dendritic PC spines through the PDZ domains of two members of the Shank family of scaffold proteins, namely Shank1 and Shank2, through which it is associated also with ITPR1, Homer proteins and AMPA receptors [74]. Via β-III spectrin, mutations in which underlie SCA5, GluRδ2 is anchored to the plasma membrane [75]. Upon intracellular calcium release that binding is disrupted and GluRδ2 gets declustered at the postsynaptic sites [76].

Moreover, GluRδ2 has been suggested to be essential for PF/PC synapse formation in the cerebellum involving trans-synaptic interaction of the N-terminal domain of the receptor via cerebellin-1 precursor protein (Cbln1, precerebellin) with presynaptic neurexins [77, 78].

### Immunohistochemistry

**Fig. 3:**
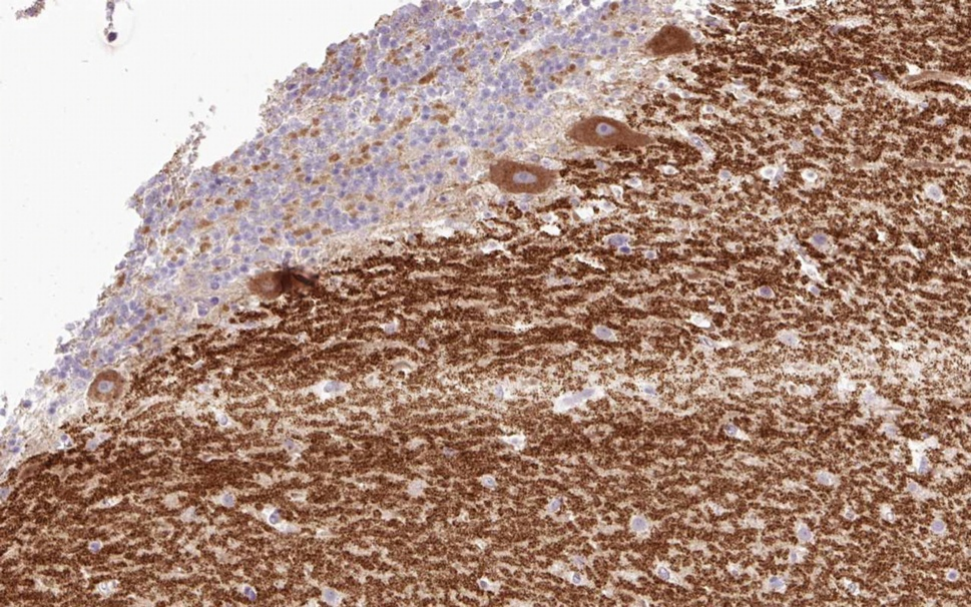
Expression of GluRδ2 in the human cerebellum as demonstrated by IHC (modified image from the *Human Protein Atlas* image database [20])

**Fig. 4:**
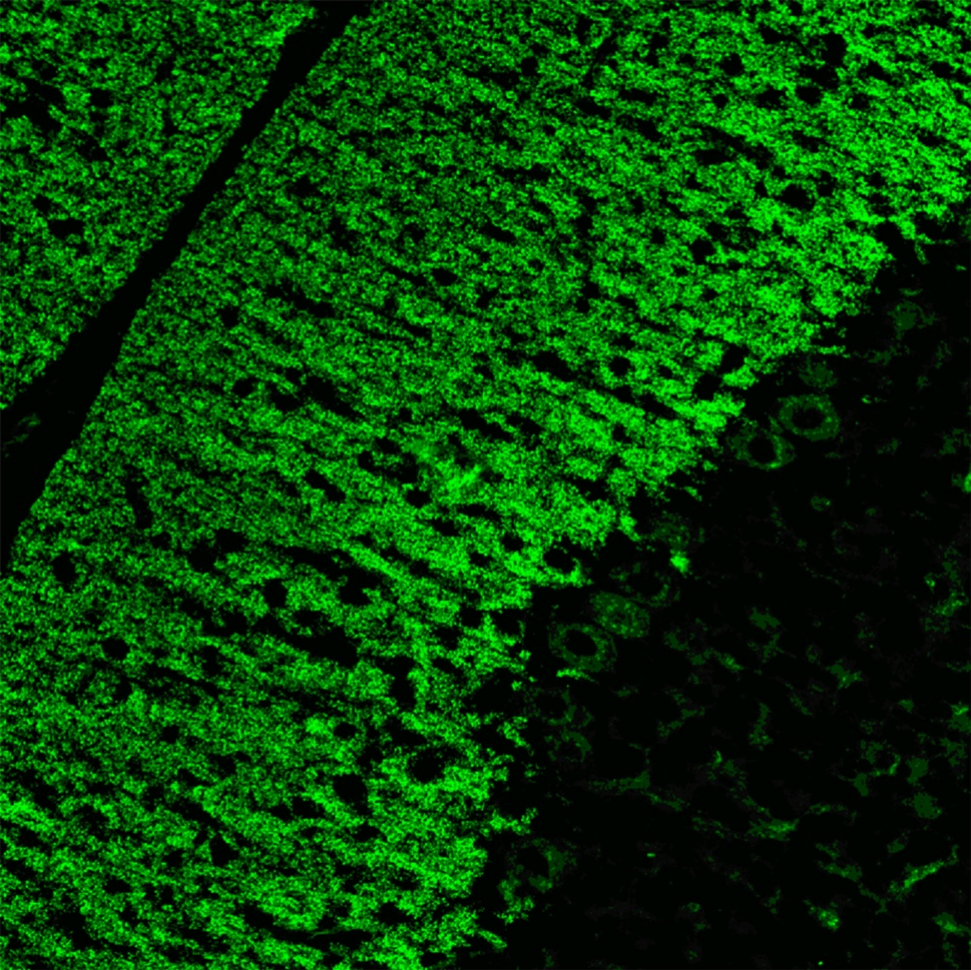
Binding of anti-GluRδ2 from a patient with ACA to a monkey cerebellum tissue section. The patient antibody was detected by use of a goat anti-human IgG secondary antibody labelled with Alexa Fluor® 488 (*green*)

GluRδ2 is thought to be selectively expressed in the cerebellum [68, 69]. It is mainly localised in the PC dendritic spines at PF/PC synapses [68, 79] (Fig. 3). As anti-GluRδ2 was originally detected by enzyme-linked immunosorbent assays (ELISA) only, no images demonstrating the IHC binding pattern of anti-GluRδ2-positive sera have previously been published. Figure 4 shows for the first time the binding of a GluRδ2-positive serum sample to cerebellum tissue.

### Antigen-specific assays

Western blot of primate cerebellum extract has been reported to contain a 62- to 80-kDa band corresponding to GluRδ2, as indicated by binding of a commercial, polyclonal GluRδ2-specific antibody, and might thus be capable of detecting anti-GluRδ2 [22]. An ELISA was used in the studies by Kinno et al. and Shimokaze et al., but no details were reported [49, 54]. Moreover, a CBA employing HEK293 transfected with human GluRδ2 (Euroimmun, Luebeck, Germany) is available at the authors’ institution for use in scientific studies.

### CSF testing

GluRδ2 antibodies were detected both in serum and in the CSF in two patients (1 × IgG, 1 × IgG and IgM), only in the serum in one, and only in the CSF in two cases.

### Association with other autoantibodies

Anti-GluRδ2 have been reported in association with anti-GluRε2 (NR2B) [51–54] and GluRζ1 (NR1) antibodies [60]. The 36-year-old woman with anti-GluRδ2 and anti-GluRε2 was also tested for antibodies to the NR1/NR2B (GluRζ1/GluRε2) heterodimer, but was negative.

### Pathogenetic relevance

A potential pathogenic role of anti-GluRδ2 in ACA is conceivable given that GluRδ2 is predominantly expressed in PCs, GluRδ2 is present on the cell surface, anti-GluRδ2 was detected in the CSF in some cases, GluRδ2 mutations are associated with ataxia (see below), and PEX had a therapeutic effect in individual patients. Of note, injection of polyclonal antibodies specific for the putative ligand-binding region of GluRδ2 generated by immunisation of rabbits with a synthetic peptide into the supracerebellar subarachnoid space was shown to cause AMPA receptor endocytosis, attenuate synaptic transmission and abrogate long-term depression in PC cultures and transient ataxia in mice [80].

### Molecular genetics

In 2013, two families were identified in which a homozygous deletion of GRID2 exon 4 and compound heterozygous deletions involving GRID2 exon 2, respectively, were associated with cerebellar ataxia, tonic upgaze and delayed cognitive and speech development. Recently, a de novo 276-kb deletion encompassing the first exon of the GRID2 gene encoding GluRδ2 was reported in a single patient, associated with cerebellar ataxia but also spastic paraplegia, frontotemporal dementia and lower motor neuron disease [81]. Finally, mutations in beta-3 spectrin underlying SCA5 were shown to affect the subcellular localisation of GluRδ2 [75]. While the receptor was enriched in the synaptosomal fractions in controls, it was not enriched in SCA5 autopsy tissue.

Additional evidence for an essential role in motor coordination comes from mutant mice experiments. Mice with a disrupted GluRδ2 locus show locomotor ataxia, disturbed PC synapse formation and impaired cerebellar LTD [82]. A recent study found that in GluRδ2 knockout mice, surplus CFs make ectopic innervations onto distal dendrites of PCs and a higher and more widespread synchrony of calcium transients in neighbouring PCs occurs. Moreover, transverse CF collaterals and CF signals transgress PC microzone borders, form ectopic synapses onto neighbouring PC dendrites and exhibit clustered firing not present in wild-type mouse [83]. Finally, mutations in the Grid2 gene have been found to be the cause of ataxia in ‘lurcher’ mice, which are characterised by apoptosis of PCs during postnatal development, and ‘hotfoot’ mice [84, 85].

## Anti-Ca/ARHGAP26

### Clinical, paraclinical and epidemiological features

Antibodies to ARHGAP26 (also termed anti-Ca) were first described in 2010 [22]. So far, four patients with Ca/ARHGAP26 antibodies have been published [22, 86, 87]. Age at diagnosis was 24, 33, 38 and 68 years. Two patients were female and two male. Recently, two additional cases have been identified by the authors (unpublished data, S.J.).

All of the patients reported thus far suffered from cerebellar ataxia, which developed subacutely within a few days in two out of the three patients with available data. The index patient presented with limb and severe gait ataxia, horizontal nystagmus, oscillopsia and dysarthria. Symptoms had developed within 5 days and had been preceded by a common cold 2 weeks before onset. Patient 2 developed a multidirectional gaze-evoked nystagmus, cerebellar dysarthria, atactic gait, severe difficulties in standing with her feet and her eyes open, and severely impaired tandem walk; 4 months before onset, she had been admitted to hospital with a 2-month history of dizziness, but MRI revealed no abnormality at that time. Patient 3 had a 9-month history of cerebellar ataxia and dysarthria, but no detailed clinical data were available. Patient 4 also presented with cerebellar gait ataxia, which progressed within a few days to panataxia with inability to walk unassisted. In addition, severe dysarthria, gaze-evoked nystagmus and disrupted vestibulo-ocular reflex cancellation with oscillopsia were noted. The patient reported slight weight loss, headaches and sweating at night, but no preceding infection or vaccination [87].

Some evidence suggests that anti-Ca/ARHGAP26 autoimmunity might possibly affect also areas outside the CNS. Interestingly, the index patient developed an increased startle response and a brisk head retraction reflex 2 months after onset, suggestive of symptomatic hyperekplexia, a condition often caused by brain stem lesions. Moreover, severe depression, restlessness and anxiety were noted, compatible with possible involvement of the limbic system.

On the other hand, occasional cases of secondary hyperekplexia due to cerebellar pathology have been reported [88], and a combination of steroid-induced and reactive depression would sufficiently explain the index patient’s psychiatric symptoms [22].

Symptoms in patient 2 and 3 included nausea and vomiting. In patient 4, cognitive decline, a flattened affect, working memory deficits, compromised verbal learning and recall, attention deficits, slowed information processing, interference difficulty and reduced spatial recognition were present in addition to a pancerebellar syndrome [87]. While some of the neuropsychological deficits could be explained by the so-called cerebellar cognitive-affective syndrome, which is characterised by impairments in visual–spatial abilities and affective disturbances such as emotional blunting and depression [89], the verbal learning and recall deficits may indicate limbic system involvement [87]. ARHGAP26 is indeed detectable in a subset of neurons in the hippocampus.

Lumbar puncture revealed CSF pleocytosis (5, 35 and 44 cells/μl; lymphocytic in two, with a few plasma cells in one; not specified in the remaining cases) and CSF-restricted OCB in all three patients tested. An increased IgG and IgM CSF/serum ratio was reported in one patient and an increased CSF/serum albumin ratio, indicating blood–CSF barrier dysfunction, in two (no data in the remainder).

Brain MRI was normal at onset in the index patient, but disclosed progressive cerebellar atrophy at month 4 and month 17 with marked widening of the cerebellar sulci and the fourth ventricle. An FDG-PET examination at month 6 revealed relative hypometabolism of the right cerebellar hemisphere. In patient 2, cerebral MRI was also unremarkable at onset but revealed cortical atrophy in the cerebellar hemispheres and the inferior vermis with enlargement of the cerebellar sulci, the fourth ventricle and the inferior cerebellar cistern when repeated at month 24; no atrophy of the cerebral cortex, midbrain or pons was noted. Patient 3 developed cerebellar atrophy, but no detailed data were reported. Patient 4 again had a normal MRI at onset but mild cerebellar atrophy was notable by month 4; repeat MRI 4 years after onset showed severe pancerebellar atrophy.

### Tumour association

Patient 2 was diagnosed with a (reportedly non-differentiated) ovarian carcinoma. Positive serum tumour markers in that patient included cancer antigen (CA)125, CA15-3 and NSE; in addition, an elevated erythrocyte sedimentation rate and increased lactate dehydrogenase blood levels were noted. At the time of first tumour diagnosis, lymph node metastasis was present. Eight months after chemotherapy, new metastases were found. Mild to moderate ARHGAP26 has been detected in numerous tumour types, including ovarian cancer, by IHC [20]. In patient 3, who is lost to follow-up, marked weight loss and fever was noted at the time of diagnosis. The remaining two patients were without malignancy at 4 and 6 years, respectively, after disease onset. Of note, in two out of the three cases with available data, onset of disease was preceded by infection.

### Outcome and prognosis

While a mild to moderate effect of steroids, rituximab and/or IVIG was noted at early stages, no such effect was observed later in the disease course. It is conceivable that the later stages reflect complete loss of Purkinje cells. In two cases with available data, rehabilitation resulted in some improvement.

In the index case, IVMP treatment with oral tapering was initially followed by marked neurological improvement. However, when steroids were reduced to 12.5 mg MP per day a significant worsening of symptoms occurred. While a further course of IVMP as well as treatment with IVIG was without effect, the cerebellar syndrome stabilised and improved following PEX and immunoadsorption. The exaggerated startle response responded to lorazepam. At last evaluation, 16 months after onset, the patient presented with cerebellar signs and hyperekplexia without significant further progression [22]. In patient 2, treatment with carboplatin and docetaxel for ovarian carcinoma and with rituximab and IVIG resulted in partial amelioration of the neurological symptoms. A relapse occurred, however, requiring double adnexectomy, hysterectomy and second-line chemotherapy with carboplatin and cyclophosphamide. Despite that treatment, symptoms continued to advance slowly and increasing gait instability, nausea and vomiting were present at last follow-up [22]. In patient 4, treatment with IVMP with oral tapering was followed by some temporary improvement; however, the patient remained unable to walk independently. Over the following 6 months, his cerebellar symptoms continued to deteriorate slowly, leaving him wheelchair-bound. While a further IVMP course in month 8 remained without effect, intensive rehabilitation was paralleled by clinical stabilisation at 12 months after onset and slight improvement at 24 months; after 3 years, the patient was able to walk with a stroller. However, at 4 years after onset his gait ataxia was gradually worsening. His affective disorder did not improve [87].

### Antigen

ARHGAP26 (also termed oligophrenin-like protein) is a GTPase-activating protein originally identified in avian cells, where it binds in an SH3 domain-dependent manner to the C-terminal domain of focal adhesion kinase (FAK), a tyrosine kinase believed to be an essential component of the integrin signalling transduction pathway regulating, inter alia, the organisation of the actin cytoskeleton upon cell–cell/cell–matrix contacts; ARHGAP26 is therefore also called GTPase regulator associated with focal adhesion kinase 1 (GRAF1) [90]. It has been shown to regulate negatively the small GTP-binding Ras homolog gene family, member A (RhoA) by stimulating its GTPase activity [90], which is involved in actin organisation, cell cycle maintenance, cellular development and transcriptional control. ARHGAP26 is a multidomain protein containing membrane-binding and -modulating Bin–amphiphysin–Rvs (BAR) and PH domains, a Rho GTPase-activating protein (GAP) domain and a dynamin- and FAK/PAK-binding SH3 domain and functions at the interface between membrane sculpting, small G-protein signalling and endocytosis [91]. It has been proposed to regulate the clathrin-independent, so-called CLIC/GEEC endocytotic pathway [92], which, by coordinating membrane remodelling into tubulovesicular carriers, mediates lipid-anchored receptor endocytosis [93] and which is able to internalise GPI-linked proteins, bacterial exotoxins and large amounts of extracellular fluid [91, 94]. These carriers are associated with the activity of cell division cycle 42 (Cdc42), a small GTP-binding protein activated by ARHGAP26 [90]. A model proposed by Doherty and Lundmark involves clustering of receptors in nanodomains of the plasma membrane that are recognised by Cdc42 (and Arf1) inside the cell; subsequently, GRAF1 is recruited to the membrane bud, resulting in the formation of a membrane tubule eventually released from the plasma membrane via a scission reaction [91].

The exact cell biological functions of this highly prevalent endocytic pathway are partly unclear. Among other functions, it has been proposed that ARHGAP26 remodels membrane microdomains at adhesion sites into endocytic carriers, facilitating membrane turnover during cell morphological changes [93], thereby giving it a role in cell spreading and migration. The biogenesis of tubular recycling endosomes (TREs), in which ARHGAP26 is involved, and their subsequent vesiculation after cargo sorting has occurred, has a role in receptor and lipid recycling to the plasma membrane [95]. In myoblasts, ARHGAP26 has been implicated in cell fusion during muscle maturation by actin remodelling and BAR-dependent membrane binding or sculpting [96].

On a subcellular level, ARHGAP26 localises to the membrane of highly prevalent, dynamic and pleomorphic tubular and punctate endocytic carriers via its N-terminal BAR and PH domains. ARHGAP26 has been shown to have effects on cell shape and actin localisation also in neuronal cells, as indicated by enhanced SPP-induced and GAP domain-dependent neurite retraction in nerve growth factor-differentiated PC12 cells overexpressing the protein [97].

Interestingly, ARHGAP26 interacts strongly with the membrane scission protein dynamin, another protein with GTPase activity [92]; accordingly, dynamin was found to co-precipitate with ARHGAP26 upon incubation with anti-Ca/ARHGAP26 [22]. Dynamin is involved also in the classical, clathrin-mediated endocytotic pathway, where it is recruited to sites of endocytosis in γ-aminobutyric acid (GABA)-ergic neurons by amphiphysin, a well-established paraneoplastic autoantigen associated with stiff-person syndrome and a broad spectrum of other neurological syndromes including progressive cerebellar degeneration [98–100]. The association of ARHGAP26 with dynamin and thus, potentially, also with the clathrin-mediated endocytotic pathway is of particular interest, since it would link ARHGAP26 to mGluR1 turnover (and thus to LTD). Agonist application has been shown to cause rapid internalisation of mGluR1 through a dynamin-dependent process [101, 102] triggered by a signalling cascade involving β-arrestin-1 [11, 103], and internalised mGluR1a has been found colocalised with clathrin in endocytic vesicles [11].

**Fig. 5:**
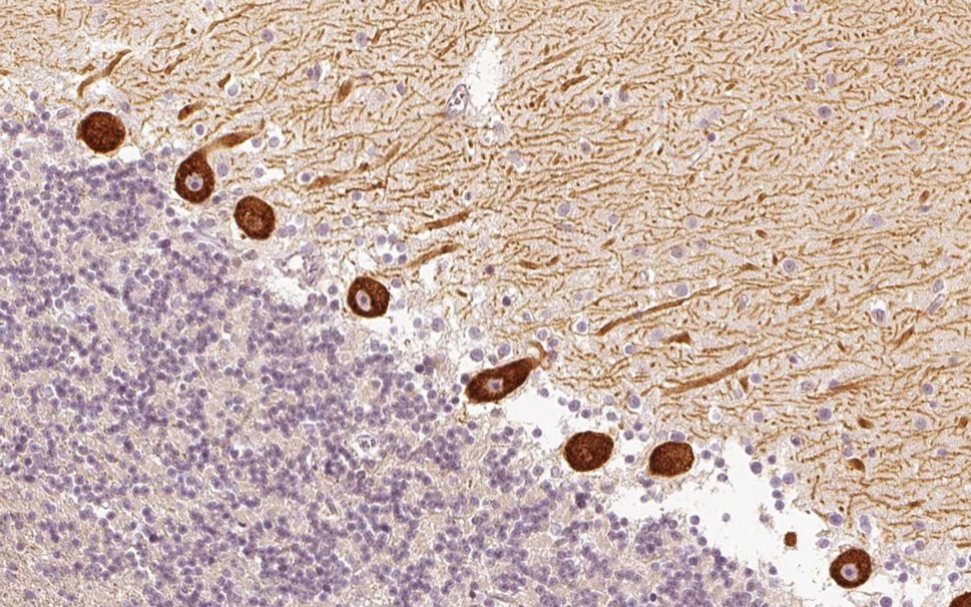
Expression of ARHGAP26 in the human cerebellum as demonstrated by IHC (modified image from the *Human Protein Atlas* image database [20])

Interestingly, PKN-beta, a fatty acid-activated serine/threonine protein kinase, has been shown to interact both with ARHGAP26 and with paraneoplastic cerebellar degeneration 17 (PCD17), or CDR2, the target antigen precipitated by anti-Yo-positive sera from patients with ACA (see the section entitled Anti-Yo/CDR2/CDR62 (PCA1)’ in Part 3 of this article series) [104, 105]. Figure 5 shows the expression pattern of ARHGAP26 in the human cerebellum.

### Immunohistochemistry

**Fig. 6:**
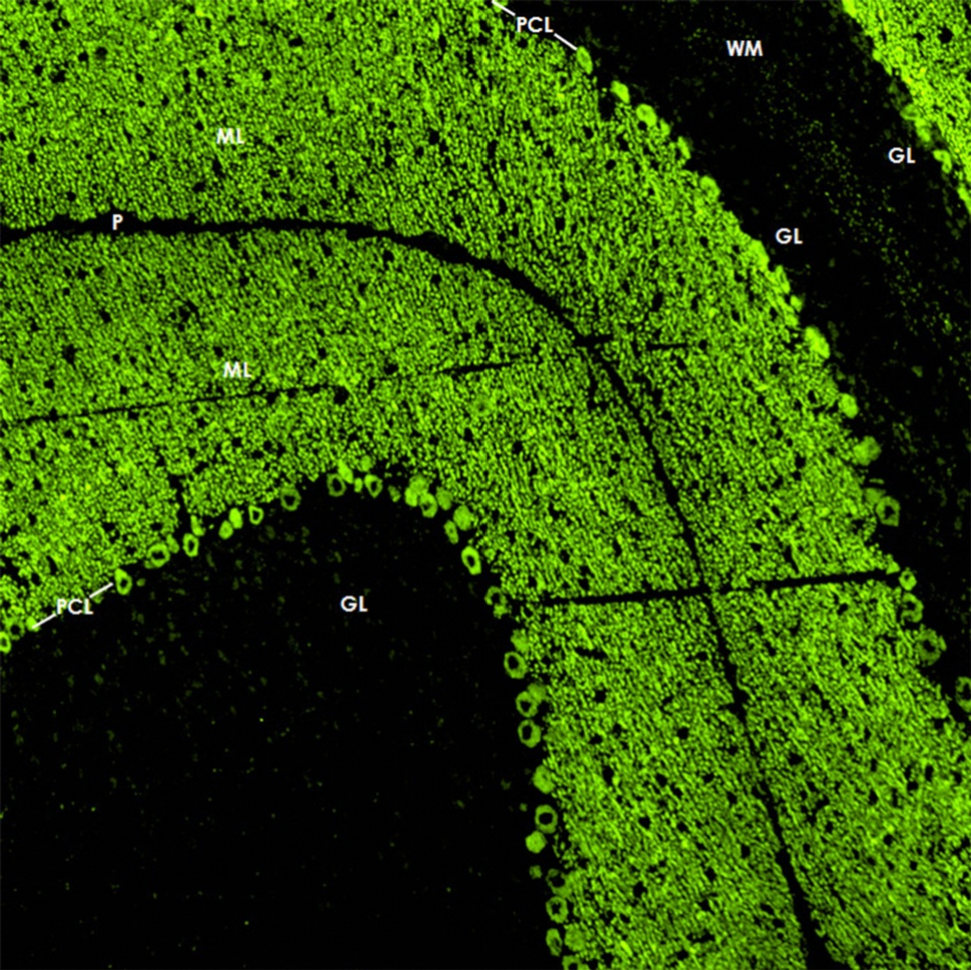
Binding of IgG from a patient with ARHGAP26-Ab-positive ACA to a mouse cerebellum tissue section. An Alexa Fluor® 488-labelled goat anti-human IgG antibody (*green fluorescence*) was used to visualise bound patient IgG. *ML* = molecular layer, *PCL* = Purkinje cell layer, *WM* = white matter, *GL* = granular layer, *P* = pia mater. Image taken from Jarius et al. [22]

When tested on snap-frozen cerebellum sections, anti-Ca/ARHGAP26 antibodies bind to the somata, dendrites (including spines) and axons of PCs (Fig. 6). Usually, the fluorescence intensity of the PC soma and axon staining is less pronounced than that of the dendritic tree. Stellar cells, basket cells, Golgi cells and the granular cells as well as their processes (including the parallel fibres) are all spared, as are the glial cells of the cerebellum and their processes. On intestinal tissue sections, binding to the plexus myentericus has been noted with high-titre samples. Incubation of hippocampal sections with anti-Ca/ARHGAP26-positive sera has resulted in staining of single neurons; however, as not all sera seem to bind to hippocampal neurons, the significance of this finding is unknown.

In accordance with their target antigen’s intracellular location, anti-Ca/ARHGAP26 did not bind to non-permeabilised cultured, live PCs but only to cultured PCs fixed and treated with CHAPS or Triton X [22]. However, the antibodies can be readily detected by IHC using non-fixed/non-permeabilised tissue if very thin sections (e.g. 5–7 μm) are used, since such sections mostly contain dissected neurons.

### Antigen-specific assays

A dot–blot assay, a preadsorption IHC assay and a HEK293-CBA (Euroimmun), all of which employ recombinant human ARHGAP26, are available at the authors’ institutions for use in scientific studies. When tested in a commercial cerebellum Western blot assay, anti-Ca/ARHGAP26-positive serum and CSF samples bound to an 80- to 97-kDa band [22].

### CSF testing

Anti-Ca/ARHGAP26 antibodies have been detected in the CSF of the only two patients examined [22]. In both cases, intrathecal synthesis as indicated by an elevated ARHGAP26-specific antibody index was noted [22]. Based on these limited data, testing of serum samples, which yielded higher titres than CSF in those two cases, may be sufficient. However, testing of the CSF may be a promising option if serum testing is hampered by non-specific background issues and/or the presence of additional autoantibodies.

### Association with other autoantibodies

Additional systemic autoantibodies (anti-nuclear antibodies [ANA] reacting with coilin) and anti-thyroidal antibodies (anti-thyroperoxidase, anti-TSH receptor) were present in two cases with available data, suggesting a more general autoimmune predisposition. However, no additional anti-neuronal antibodies were found.

### Pathogenetic relevance

A potential pathogenic role is suggested by the fact that the antibody is usually present at high titres, is produced intrathecally, belongs to the IgG1 subgroup and was associated with PCD in all cases reported thus far. However, ARHGAP26 is believed to be an intracellular antigen, rendering it possible that the actual damage is caused by T cells rather than by the antibody itself. Passive transfer experiments are currently under way.

Of note, anti-coilin ANA were present in the index patient. P80 coilin has been reported to interact with and possibly alter the function of ataxin-1, a protein involved in SCA1. However, anti-coilin ANA are not normally associated with ACA, rendering it a priori unlikely that these antibodies contributed to that patient’s neurological condition. Moreover, anti-coilin ANA were absent in all other published patients and in all subsequent cases, as yet unpublished, diagnosed at our laboratory.

### Molecular genetics

ARHGAP26 has not been implicated in SCA to date. However, somatic mutations of ARHGAP26 have been identified as a rare cause of juvenile myelomonocytic leukaemia, one of the most common paediatric myelodysplastic syndromes [106]. The ARHGAP26 gene is highly homologous to BCR, which is also involved in a leukaemia-associated translocation [90]. Recently, decreased expression of ARHGAP26 has been implicated in the pathogenesis of X-linked alpha-thalassaemia mental retardation syndrome [107]. GRAF1 knockout mice developed normally, were fertile and were of similar weight as wild-type animals; haematoxylin and eosin-stained cerebellum and hippocampus sections showed no gross anatomical abnormalities, though detailed neurological or electrophysiological examinations were not performed [108].

## Anti-VGCC

### Clinical, paraclinical and epidemiological features

Anti-VGCC autoantibodies have been reported both in patients with PCD and in patients with non-paraneoplastic cerebellar ataxia. In addition, they exist both in tumour-associated and in non-paraneoplastic Lambert–Eaton myasthenic syndrome (LEMS).

Graus et al. reported 16 anti-VGCC-positive male patients with PCD and no CNS symptoms beyond cerebellar ataxia [109]. No detailed data on the patients’ cerebellar symptoms were provided. Nine of the 16 had no clinical evidence of LEMS at the time of anti-VGCC testing. No information on MRI and LP results was provided [109].

Bürk et al. found anti-P/Q-type VGCC antibodies in four female and four male patients with non-paraneoplastic cerebellar degeneration. Additional symptoms besides ataxia, dysarthria and cerebellar oculomotor deficits included cognitive symptoms, spasticity, impaired proprioception, double vision, dysphagia, autonomic dysfunction and basal ganglia symptoms [110]. Four patients had previously been diagnosed with idiopathic multiple system atrophy of the cerebellar type and four with idiopathic late-onset ataxia. MRI showed cerebellar atrophy in six out of eight and olivopontocerebellar atrophy in the remainder. CSF results were not reported.

### Tumour association

First reports on the association of lung cancer, cerebellar degeneration and myasthenic symptoms date back to the 1970s [111]. A role of antibodies to VGCC in patients with PCD was first suggested by Clouston et al., who reported three PCD patients with SCLC, small-cell prostate cancer or non-Hodgkin lymphoma, respectively, two of whom had in addition LEMS [112]. Shortly after, Lennon et al. reported both P/Q- and N-type VGCC antibodies in an independent cohort [113]; however, no detailed data on the frequency among the various underlying tumours were provided, and almost all of the PCD patients included (*n* = 34) had additional anti-neuronal antibodies. In a third study, which comprised only patients with small-cell lung cancer (SCLC) and PCD, anti-P/Q-type VGCC autoantibodies were detected in 12/50 cases (24 %) using a ^125^I-ω-conotoxin MVIIC-based assay, including all patients with signs or symptoms of LEMS but also five PCD patients without LEMS [114]. By contrast, only 1 of 49 control patients with SCLC but no paraneoplastic CNS syndrome had P/Q-type VGCC antibodies. Similarly, antibodies to N-type VGCC were detected in the same study using a ^125^I-ω-conotoxin GVIA-based radioimmunoprecipitation assay (RIA) in 9/50 samples from SCLC-PCD patients, six of whom (66 %) had no LEMS at the time of testing. Voltz et al. [115] used a commercial RIA based on ^125^I-ω-conotoxin MVIIC to detect anti-VGCC in eight out of 66 patients with SCLC and anti-Hu syndrome. Of these eight patients, two had PCD without LEMS and two PCD with LEMS. Of note, none of 27 patients with PCD and tumours other than SCLC was positive for P/Q-type VGCC antibodies. By contrast, P/Q VGCC antibodies were absent in a smaller series of anti-Yo-positive patients with gynaecological tumours [116]. Graus et al. examined PCD patients with lung cancer with or without anti-Hu antibodies (23 %) but with no symptoms beyond the cerebellum for anti-P/Q-type VGCC, again using a ^125^I-ω-conotoxin MVIIC assay, and detected raised levels in 16 (41 %) of 39 patients with positive PCD, 9 of whom did not have clinical or subclinical LEMS (56 %) and 14 of whom were negative for anti-Hu [109]. The median time between onset of PCD and tumour diagnosis was 3 months. The most frequent lung tumour diagnosis was SCLC; only two patients had NSCLC. Except for the prevalence of LEMS, no relevant differences were observed between PCD patients with and without VGCC antibodies. The authors concluded that anti-P/Q-type VGCC could be a more sensitive marker for PCD than anti-Hu and in general a useful marker for lung cancer-associated PCD.

However, anti-VGCC autoantibodies are not always associated with cancer in patients with cerebellar ataxia. Clouston et al., in their original series on anti-VGCC in ataxia patients, reported two non-paraneoplastic cases [112], and Bürk et al. found P/Q-type VGCC-specific antibodies in 8/67 (12 %) of patients with non-paraneoplastic chronic cerebellar degeneration; by contrast, sera from 117 control patients were negative in the latter study [110].

### Outcome and prognosis

Outcome and prognosis may depend on tumour diagnosis. Graus et al. reported 16 patients with SCLC and anti-VGCC-PCD, 8 of whom already had a Rankin score of >3 at first presentation. Nine received tumour treatment and immunotherapy, four only immunotherapy, and three no treatment. One patient improved, five stabilised at a low Rankin score (≤3), and five worsened or stabilised at a high Rankin score (no data for the remainder). Median survival time was 12 months. Interestingly, in the three patients with both PCD (anti-Hu status not reported) and LEMS, only LEMS improved following immunotherapy.

A patient with ataxia and anti-VGCC but no additional antibodies reported by McKasson et al. improved under treatment with IVIG, prednisone and mycophenolate mofetil but deteriorated after IVIG was halted because of aseptic meningitis [117]. After developing LEMS 11 months later, the patient declined further treatment and elected hospice care.

Anti-neuronal antibodies are thought to improve survival from SCLC [118]. However, in SCLC patients without neurological symptoms, no effect of anti-VGCC on extent of disease or survival was found in a study comparing 10 anti-VGCC-positive with 143 anti-VGCC-negative patients [119].

Two patients with anti-VGCC autoantibodies but non-paraneoplastic PCD were still alive 10 and 2 years after onset [110]. Rigamonti et al. reported a patient with non-paraneoplastic PCD and subclinical LEMS with high serum and CSF VGCC titres in whom IVIG treatment led to clinical improvement and reduction of serum antibody titre over a 13-month follow-up period [120].

### Antigen

Calcium channels are the main molecular link between cellular membrane potential changes and intracellular Ca^2+^ signalling and can be divided into ligand-gated and voltage-gated channels. Upon activation, they allow Ca^2+^ from the extracellular space or from intracellular calcium stores to enter the cytosol. VGCCs, which are membrane proteins, are further subclassified according to subunit composition, voltage dependence and single-channel conductance kinetics. P/Q-type VGCCs are dihydropyridine-insensitive high-voltage threshold-activated (i.e. they require strong membrane potential depolarising stimuli for activation) cation channels mediating so-called P- and Q-type Ca^2+^ currents [121] and were first identified in PCs. The channels are made up of the principal pore- and gating apparatus-forming as well as plasma membrane voltage-sensing subunit termed alpha-1A (Ca_v_2.1), which is encoded by the CACNA1A gene, and the auxiliary channel-trafficking and activity-regulating subunits beta, alpha-2/delta (disulphide-linked), and, in some tissues, gamma [122, 123]. They are blocked by spider and marine snail peptide toxins, in particular by ω-agatoxin IVA and ω-agatoxin IVB from funnel web spider venom which bind with high specificity and affinity [124].

P/Q-type VGCCs autoantibodies play an important role in glutamatergic neurotransmission by being involved in spatiotemporal Ca^2+^ regulation. They are particularly important for Ca^2+^-dependent neurotransmitter and hormone vesicle release at central and peripheral synaptic terminals, though many other functions have been described [125–130].

Of note, Ca_v_2.1 and mGluR1a have been shown to be co-localised at the PC dendritic plasma membrane and to interact directly via their C-terminal intracellular domains, forming a heteromeric protein complex [131–133]. MGluR1 is thought to inhibit Ca_v_2.1-mediated Ca^2+^ currents both in a ligand-independent manner mediated by physical coupling to Ca_v_2.1 and in a ligand-dependent manner mediated by G_i_/G_o_ coupling [131, 134, 135]. By contrast, simultaneous stimulation of mGluR1 and Ca_v_2.1 induces large Ca^2+^ increases [131].

### Immunohistochemistry

**Fig. 7:**
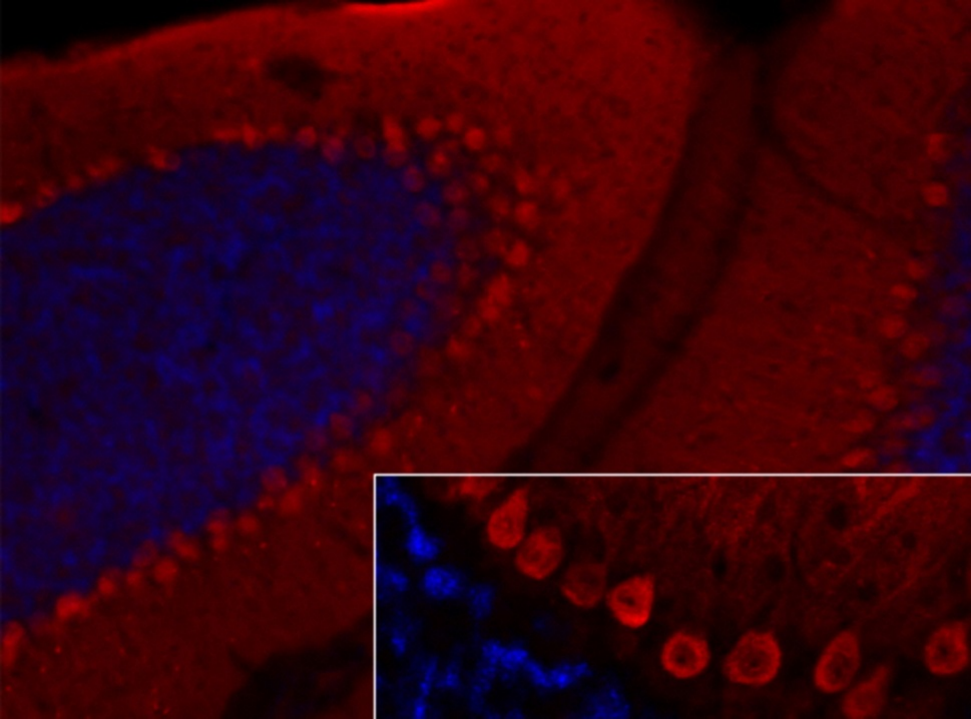
Expression of Ca_v_2.1 in the mouse cerebellum as detected by IHC (modified image from Mallmann RT et al. Ablation of Ca(V)2.1 voltage-gated Ca^2+^ channels in mouse forebrain generates multiple cognitive impairments. PLoS One 2013 Oct 31;8(10):e78598 doi: 10.1371/journal.pone.0078598. © 2013 Mallmann et al.). Note that IHC is not an established method for the detection of anti-VGCC autoantibodies, which are usually determined by RIA

So far, no images showing binding of IgG from anti-VGCC-positive patients to cerebellum tissue sections have been published. However, by means of polyclonal antibodies generated from isolated P-type channels, Hilman et al. could demonstrate that P-type VGCCs are present on the plasma membrane of PC somata and PC main stems and, at much higher levels, on that of the PC dendrites, spiny branchlets and spines [136]. Electron microscopy demonstrated expression also in the PC ER. Figure 7 shows staining of PC somata and dendrites by a polyclonal antibody to Ca_v_2.1.

Outside the cerebellum, P-type VGCC autoantibodies are abundantly expressed throughout the CNS [136–138]: Strong expression was found in the periglomerular cells of the olfactory bulb, scattered neurons in the deep layer of the entorhinal and pyriform cortices, neurons in the brainstem, habenula, nucleus of the trapezoid body and inferior olive and along the floor of the fourth ventricle [136]. Medium-intensity reactions were observed in layer H pyramidal cells of the frontal cortex, the CA1 cells of the hippocampus, the lateral nucleus of the substantia nigra, lateral reticular nucleus and spinal fifth nucleus. Light labelling was seen in the neocortex and striatum [136].

Indirati et al. described a somatodendritic gradient of P/Q-type channels, with the density increasing 2.5-fold from soma to distal dendrites [139], and co-clustering of a subpopulation of Ca_v_2.1 with the calcium-activated potassium channels BK and SK2 in the PC somata and primary dendrites [139].

Ca_v_2.1 co-localises with mGluR1 mainly in the molecular layer of the cerebellum (except for the interneuron somata), whereas only weak staining was seen in the PC layer [131].

Q-type VGCCs are found in cerebellar granule neurons and the hippocampus.

### Antigen-specific assays

All studies published so far used ^125^I-ω-conotoxin-based RIAs employing native VGCC from human or non-specified homogenised cerebellum tissue extract. There are some potential limitations. First, ^125^I-ω-conotoxin MVIIC may not be completely specific for Ca_V_2.1 (α1A, P/Q-type) channels; it has also been reported to block Ca_v_2.2 (α1B, N-type), albeit with lower affinity. Similarly, ^125^I-ω-conotoxin GVIA might possibly label also P2X receptors. Second, subclinical LEMS had not been excluded by electrophysiological studies in all patients, and some indeed later developed clinical or electromyographic features of LEMS. Establishing recombinant assays for VGCC has turned out to be challenging; so far, no CBA or Western blot has been reported. Also, no RIA that would differentiate between P- and Q-type VGCC antibodies exists to date. Assays using omega-agatoxin IVA, a P-type specific blocker, are currently under development.

### CSF testing

In the study by Graus et al., 7/15 CSF samples had anti-P/Q-type VGCC, with evidence of intrathecal synthesis as determined by calculation of the anti-VGCC-specific antibody index in four [109]. Intrathecal synthesis was also suggested by very high CSF anti-VGCC concentration compared to serum in another study [112].

### Association with other autoantibodies

In SCLC patients with PCD, anti-VGCC have been reported in association with anti-Hu [109, 113–115], anti-Yo [113, 140], anti-Zic4 [141] and, with particularly high frequency, SOX1 antibodies [141]. Bürk et al. found no association of anti-P/Q VGCC with Hu, Ri, Yo or amphiphysin in 67 patients with idiopathic cerebellar degeneration [110].

While two studies detected antibodies against VGCC subunit other than alpha-1 in a subset of patients with LEMS [142–144], no reactivity to the β4 or γ2 auxiliary subunits of VGCC was found in sera from PCD patients in a HEK293 CBA [141]. However, the authors of the latter study identified in a single patient with SCLC-associated PCD and anti-P/Q-type VGCC a novel autoantibody to the cytoskeletal matrix of the nerve terminals active zone (CAZ) protein ELKS1 (also termed ERC1) [141], which was recently shown to interact and co-localise with the auxiliary β4 subunit of VGCC [145]. In parallel, the same antigen was identified by another group in patients with LEMS [146].

### Pathogenetic relevance

Anti-VGCC autoantibodies are known to be pathogenic in LEMS [147]. However, there is also strong evidence for a pathogenic role of anti-P/Q-type VGCC in PCD, in line with the fact that P/Q-type VGCCs are present on the cell surface and thus accessible to circulating IgG. Binding of anti-VGCC to live PCs was indeed demonstrated using cerebellar slice culture and PC culture models [117, 148]. Most importantly, Martín-García et al. reported that intrathecal injection of IgG purified from the serum of a P/Q-type VGCC antibody-positive patient with PCD and LEMS (but not IgG from controls) induced marked, reversible ataxia in mice [30]. As no human complement was co-injected and symptoms were reversible, it is most likely that the effect was caused by functional blocking of VGCCs.

In an earlier study, Liao et al. had demonstrated that injection of a polyclonal peptide antibody against a major immunogenic region in P/Q-type VGCCs (the extracellular domain-III S5–S6 loop) was capable of inhibiting VGCC function in neuronal and recombinant VGCCs, altering cerebellar synaptic transmission by attenuating Ca^2+^ currents and causing cerebellar ataxia in mice [149].

Fukuda et al. found a reduction in the quantity of cerebellar P/Q-type VGCC in human autopsy cerebellar tissues from three PCD/LEMS patients compared with tissues from six disease controls, including one with LEMS but no PCD; the decrease was most pronounced in the molecular layer [150]. In line with a contribution of anti-VGCC to this finding, the ratio of autoantibody-VGCC complexes to total P/Q-type VGCCs in PCD/LEMS tissue was increased in these patients, as demonstrated by autoradiography using ^125^I-ω-conotoxin MVIIC.

The absence of PCD in many anti-VGCC-positive patients with LEMS and the absence of LEMS in some PCD patients with high antibody titres are not well understood. Of note, intrathecal injection of IgG from a patient with anti-P/Q-type VGCC and LEMS but not PCD did not induce ataxia in mice in the above-cited study by Martín-García et al. [30]. Together, this could indicate that differences in anti-VGCC fine specificity play a role. However, alternative explanations such as site-specific compensatory upregulation of other VGCC subtypes cannot be ruled out [109].

The VGCC subtype specificity was investigated by Pinto et al., who demonstrated that IgG antibodies to presynaptic VGCCs at motor nerve terminals from LEMS patients caused a significant dose-dependent reduction in the K^+^-stimulated Ca^2+^ increase in HEK293 cells expressing the alpha1A subunit of P/Q-type VGCC but not in cells expressing the N-type VGCC-defining alpha1B subunit (Ca_v_2.2), the L-type VGCC-related alpha1D or alpha1C subunits (Ca_v_1.2 and Ca_v_1.3, respectively), or the R-type-defining alpha1E subunit (Ca_v_2.3) [148]. In line with this finding, incubation of cultured rat cerebellar neurons with LEMS IgG was followed by a reduction in P-type current in Purkinje cells and in both P- and Q-type currents in granule cells [148].

A pathogenic role of the antibody is further supported by the finding of CSF positivity and even intrathecal anti-VGCC synthesis in patients with VGCC-PCD [109] and by molecular findings indicating that malfunction of VGCC causes hereditary ataxia (though polyglutamine-mediated cell toxicity plays a major role as well in some of the latter cases). Whether other factors in addition to antibody-mediated mechanisms, such as T-cell-mediated mechanisms, play a role is as yet unknown.

### Molecular genetics

Different mutations of the CACNA1A genes, which encode the immunogenic alpha-1A VGCC subunit (Ca_v_2.1), can produce the autosomal dominant SCA type 6, episodic ataxia type 2 (EA2) or familial hemiplegic migraine 1 (FHM1). SCA6 is characterised by initially mild but slowly progressive gait and limb ataxia with dysarthria, decreased muscle tonus, horizontal and vertical gaze nystagmus (often presymptomatic), abnormal vestibulo-ocular reflex, cerebellar atrophy affecting the vermis more than the hemispheres with extensive loss of PCs and proliferation of Bergmann glia [151–156]; additionally, in rare patients, neuropathy [157, 158] is caused by an expanded CAG(n) repeat in the C-terminal coding region of CACN1A [152, 154].

Two mutations causing a disrupted reading frame [159], two splice mutations in the CACNA1A gene [160], several (partly large-scale and partly exonic) deletions [161, 162] and a duplication in CACNA1A [162] have been identified as causes of EA2. EA2 is an autosomal dominant disorder with onset in childhood or early adolescence that is characterised by paroxysmal attacks of ataxia, vertigo and nausea responsive to acetazolamide. Episodes can last from minutes to days and can be associated with, among other symptoms, hemiplegia and headache, including migraine. Cerebellar atrophy affecting predominantly the vermis [163] and persistent, attack-independent symptoms of ataxia may develop.

Finally, cerebellar atrophy and persisting ataxia occur also in FHM1, an autosomal dominant disorder causing migraine with hemiplegic (and at least one other) aura symptoms. FHM1 is caused by missense mutations in the P/Q-type VGCC gene in 50 % of affected families, including all of those with permanent cerebellar symptoms [159, 164, 165].

Similarly, in mice several spontaneous or induced mutations have been described that further underline the high importance of VGCC for proper cerebellar function [166–169]. Mice lacking P/Q-type VGCC are ataxic and dystonic, have absence seizures and die within 3 weeks [166]. A missense mutation in the alpha1A VGCC subunit affecting the pore lining region and leading to a reduction in channel function was shown to underlie ataxia (and seizures) in the ‘tottering’ mouse [169, 170]. In the homozygous ‘leaner’ mouse, a substitution in the VGCC alpha1A gene causing splicing failures results in reduced P-type calcium currents predominantly in PCs and severe ataxia accompanied by significant loss in PC numbers [169, 171, 172].

## Note to the reader

In the following third part of this series, we will review the current knowledge on anti-Tr/DNER-, anti-Nb/AP3B2-, anti-Yo/CDR2- and PCA-2-associated ACA, discuss diagnostic pitfalls and give a summary and outlook [173].

## Abbreviations

Aa: amino acids
ACA: autoimmune cerebellar ataxia
AChR: acetylcholine receptor
ADEM: acute disseminated encephalomyelitis
AF: Alexa Fluor®
AGNA: anti-glial cell nuclear antibodies
AMPA: α-amino-3-hydroxy-5-methyl-4-isoxazolepropionic acid
ANA: anti-nuclear antibodies
ANNA: anti-neuronal nuclear antibody
APTX: aprataxin
AQP4: aquaporin-4
ARHGAP26: Rho GTPase-activating protein 26
ATXN1: ataxin-1
BAR: Bin–amphiphysin–Rvs
beta-NAP: neuronal adaptin-like protein
CARP VIII: carbonic anhydrase-related protein VIII
CASPR2: contactin-associated protein-2
CBA: cell-based assay
cDNA: complementary DNA
CDR2: cerebellar degeneration-related protein 2
CDR2L: CDR2-like
CDR3: cerebellar degeneration-related autoantigen-3
CF: climbing fibre
CHO: Chinese hamster ovary
CNS: central nervous system
CSF: cerebrospinal fluid
CTL: cytotoxic T lymphocytes
DAG: diacylglycerol
DAPI: 4′,6-diamidino-2-phenylindole
DNA: deoxyribonucleic acid
DNER: delta notch-like epidermal growth factor-related receptor
DPPX: dipeptidyl-peptidase 6
EA2: episodic ataxia type 2
EGF: epidermal growth factor
ELISA: enzyme-linked immunosorbent assay
ER: endoplasmic reticulum
FHM1: familial hemiplegic migraine 1
FITC: fluorescein isothiocyanate
GABA: γ-aminobutyric acid
GABABR: GABA type B receptor
GAD: glutamic acid decarboxylase
GAP: Rho GTPase-activating protein
GFAP: glial fibrillary acidic protein
GL: granular layer
GluR: ionotropic glutamate receptors
GluRδ2: glutamate receptor delta-2
GRAF1: GTPase regulator associated with focal adhesion kinase 1
HEK: human embryonic kidney
IB: immunoblot assay
ICC: immunocytochemistry
IIF: indirect immunofluorescence
IgA: immunoglobulin A
IgG: immunoglobulin G
IgM: immunoglobulin M
IHC: immunohistochemistry
IP: immunoprecipitation assay
IP3: inositol 1,4,5-trisphosphate
IRBIT: IP_3_ receptor-binding protein released with IP3
ITPR1: inositol 1,4,5-trisphosphate receptor, type 1
IVIG: intravenous immunoglobulins
IVMP: intravenous methylprednisolone
kDa: kilodalton
LEMS: Lambert–Eaton myasthenic syndrome
LGI1: leucine-rich, glioma inactivated 1
LTD: long-term depression
LTP: long-term potentiation
mGluR1: metabotropic glutamate receptor 1
ML: molecular layer
MRI: magnetic resonance imaging
mRNA: messenger RNA
NMDA: *N*-methyl-d-aspartate
NMO: neuromyelitis optica
NSCLC: non-small cell lung cancer
NSE: neuron-specific enolase
OCB: oligoclonal bands
PC: Purkinje cell
PCA: Purkinje cell antibody
PCD: paraneoplastic cerebellar degeneration
PCL: PC layer
PF: parallel fibres
PIP_2_: phosphatidyl 4,5-bisphosphate
PKCγ: protein kinase C gamma
PLCβ3: phospholipase Cβ3
PSD: postsynaptic density
RACK: receptors for activated C kinases
RhoA: Ras homolog gene family, member A
RIA: radioimmunoprecipitation assay
RNA: ribonucleic acid
SCA: spinocerebellar ataxia
SCLC: small-cell lung cancer
SOX: sex-determining region Y-box
TRPC: canonical transient receptor potential channel
TUNEL: TdT-mediated dUTP-biotin nick end labelling
VGCC: voltage-gated potassium channel
VGKC: voltage-gated potassium channel
WB: Western blot
WM: white matter
